# The research landscape and future of targeting super-enhancers for cancer therapy: a bibliometric analysis

**DOI:** 10.1007/s12672-026-04471-w

**Published:** 2026-01-28

**Authors:** Wenhao Sun, Yupeng Hu, Tianjun Lan, Yingnan Ma, Yupeng Wu, Chaobin Pan, Lianxi Mai, Zhaoyu Lin

**Affiliations:** 1 Guangdong Provincial Key Laboratory of Malignant Tumor Epigenetics and Gene Regulation, Sun Yat-Sen Memorial Hospital, Sun Yat-sen University, Guangzhou, China; 2 Department of Oral & Maxillofacial Surgery, Sun Yat-Sen Memorial Hospital, Sun Yat-sen University, Guangzhou, China

**Keywords:** Bibliometric analysis, Super-enhancer, Targeting therapy, Cancer, Landscape

## Abstract

**Background:**

Super-enhancers are specialized transcriptional regulatory elements pivotal for establishing and maintaining cell identity. Since their identification in 2013, SEs have garnered considerable attention as promising therapeutic targets in oncology. However, despite substantial progress, the molecular mechanisms and translational potential of SE-targeted strategies are not fully systematized, necessitating a comprehensive bibliometric analysis to map the intellectual landscape and guide future research.

**Methods:**

We conducted a bibliometric analysis of 928 publications (2013–2024) from the Web of Science Core Collection using VOSviewer, CiteSpace, and R. The study employed co-occurrence, co-citation and cluster analysis to profile the research landscape, identifying leading contributors, influential works, and conceptual themes. Temporal analysis and burst detection were further applied to track evolution and pinpoint emerging frontiers in super enhancer-targeted cancer therapy.

**Results:**

The intellectual architecture is dominated by the United States and China, with Harvard Medical School and Shanghai Jiao Tong University as pivotal institutions and Young Richard A. as the most influential author. Temporal mapping revealed a progression from core themes like “gene expression” and “selective inhibition” to contemporary foci on “drug resistance” and the nascent frontier of “enhancer RNA”, signaling a collective shift from basic biology to therapeutic innovation.

**Conclusion:**

This study consolidates a decade of progress in super enhancer-targeted cancer therapy, mapping its evolution from mechanistic discovery to translational ambition. By pinpointing emergent frontiers such as enhancer RNA, our analysis provides a strategic roadmap to guide future research and accelerate the clinical translation of super enhancer-directed strategies, ultimately aiming to overcome persistent therapeutic barriers such as drug resistance in oncology.

**Supplementary Information:**

The online version contains supplementary material available at 10.1007/s12672-026-04471-w.

## Introduction

Cancer remains one of the most severe global health challenges, profoundly impacting patients’ physical and psychological well-being and imposing a substantial economic burden worldwide [1]. In 2023 alone, an estimated 18. 5 million new cancer cases (95% uncertainty interval:16.4–20.7; excluding non-melanoma skin cancer) and 10. 4 million cancer-related deaths (9.6–10.9 million) were reported globally [2, 3]. Tumorigenesis typically originates from genetic alterations in hereditary material, leading to dysregulation between oncogenes and tumor suppressor genes and ultimately resulting in uncontrolled cellular proliferation [4]. Current therapeutic modalities, including surgery, radiotherapy, chemotherapy, immunotherapy, and their combinations in neoadjuvant or adjuvant settings, aim to eliminate malignancies or control their progression [5]. However, each approach presents significant limitations. Surgical resection often causes substantial tissue trauma and functional impairment, and is unsuitable for certain cancers such as recurrent nasopharyngeal carcinoma [6] or disseminated metastatic cancer [7]. Radiotherapy and chemotherapy exhibit limited efficacy in treatment insensitive cases and frequently cause unnecessary toxic exposure and side effect to healthy tissues [8]. Moreover, drug resistance commonly develops during cytotoxic chemotherapy exploiting the defective of a cancer cell, such as impaired DNA repair mechanisms, to preferentially drive cancer cells to programmed cell death [9]. In the aspect of immunotherapy such as with PD-1/PD-L1 blockade, several limitations remain evident, including poor tissue penetration, an inability to cross physiological barriers like the blood-brain barrier, lack of oral bioavailability, immunogenicity, immune-related adverse events (irAEs), limited access to intracellular targets, and sex-based efficacy disparities [10–14]. These challenges underscore the urgent need to develop precise and efficient targeted therapies tailored to specific cancer types and stages.

Super-enhancers (SEs) represent a specialized class of transcriptional regulatory elements, formed by the dense clustering of classical enhancers that are co-occupied by master transcription factors and mediator complexes at key cell identity genes [15]. Functioning as potent cis-regulatory modules, SEs play a pivotal role in establishing and maintaining cell identity. The concept was first introduced in 2013 by the team of Richard A. Young, who demonstrated that SEs govern cell type-specific gene expression programs [16]. Compared with typical enhancers, SEs exhibit several distinguishing characteristics. Firstly, they span significantly larger domains, typically extending 8–20 kilobases in length [1]. Secondly, SEs recruit a higher density of transcriptional factors and co-activators(such as BRD4 and CDK7), and exhibit enriched histone modifications, most notably H3K27ac, which serves as a canonical marker for their identification and discrimination from classical enhancers [16–19]. As a result of these structural and compositional features, SEs significantly amplify the transcriptional output of their target genes relative to conventional enhancers [20].

SEs are closely associated in tumorigenesis due to their role in transcriptional deregulation as a hallmark of cancer-driven by alterations in both coding genes and non-coding regulatory elements [21]. By maintaining cancer cell identity and amplifying oncogenic transcription, SEs facilitate malignant proliferation, metastasis, drug resistance, and inflammatory responses. Emerging evidence indicates that SEs exert their regulatory influence not only through protein-coding genes but also via non-coding RNAs (ncRNAs), thereby modulating biological functions both directly and indirectly [22, 23]. To date, SEs have been increasingly investigated as therapeutic targets across multiple cancer types, such as prostate cancer [23], lung adenocarcinoma [20, 24], breast cancer [25], and ovarian cancer [26]. Despite this promising landscape, key challenges still remain. The molecular mechanisms and regulatory networks involving SEs are not fully elucidated, and their clinical translation is still nascent. Critical areas such as the crosstalk between SEs, tumor immunity, and epigenetic regulation, as well as the efficacy of SE-directed combination therapies, require further investigation. Against the backdrop of rapidly expanding research, a systematic analysis of the current landscape and emerging trends in SE-based tumor targeting therapy is both timely and necessary.

Bibliometric analysis has gained prominence as a powerful tool for synthesizing large volumes of academic literature, enabling the identification of research trends, collaborative networks, and emerging frontiers through quantitative examination of publications, keyword co-occurrence, and co-cited literature [27–31]. Despite its widespread application, no study to date has employed bibliometric methods to systematically investigate the landscape of SE research in the context of cancer targeting therapy. To address this gap, we retrieved relevant publications from the Web of Science Core Collection(WoSCC) database spanning 2013 to 2024 and conducted a comprehensive bibliometric analysis using VOSviewer, CiteSpace, and R. This study aims to map the intellectual structure of the field, identify influential authors and institutions, trace thematic evolution, and highlight future research directions. To our knowledge, this work represents the first dedicated bibliometric analysis focusing specifically on SE-targeting therapeutic strategies for cancer treatment, thereby providing a distinct translational perspective that complements existing broad landscape studies.

## Materials and methods

### Data source and research strategy

Data were retrieved from the Web of Science Core Collection (WoSCC) to leverage its comprehensive and well-structured scientometric records. The publication period spanned from the inception of the database to December 31, 2024, ensuring a stable and complete dataset for analysis. The search query was formulated as follows: TS=(“tumo*” OR “cancer*” OR “carcinoma*” OR “sarcoma*” OR “neoplasm*” OR “malignant*”) AND TS=(“super-enhancer*” OR “super enhancer*”) AND TS=(“therap*” OR “treat*” OR “drug*” OR “inhibitor*” OR “target*” OR “targeted therapy” OR “pharmacological intervention” OR “therapeutic strategy” OR “small molecule” OR “epigenetic therapy”). The initial results were refined by excluding non-English publications and limiting the document types to Article and Review. This process yielded 928 relevant publications, with each record containing complete metadata, including publication year, journal, authors, affiliations, keywords, and cited references.

### Bibliometric data analysis

**Fig. 1:**
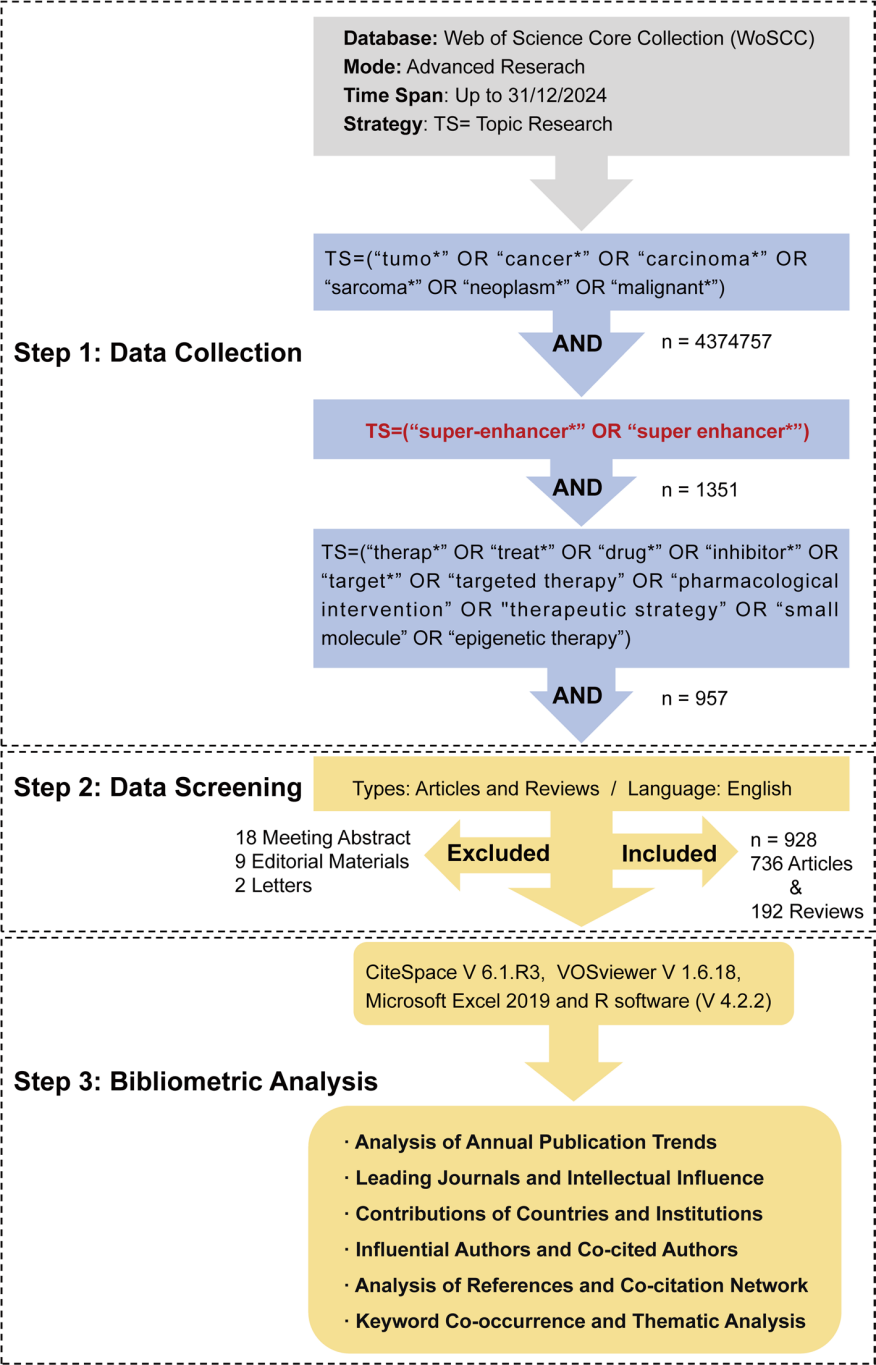
Publication strategy and data analysis workflow

The full data of all included documents were exported in plain text format. These records were then processed using a suite of analytical tools. Network mapping and burst detection were performed with CiteSpace 6.1.R3, co-occurrence analysis and visualization were conducted in VOSviewer 1.6.18, while basic data tabulation and statistical calculations were handled in R software (version 4.2.2). The detailed literature collection and screening workflow is presented in Fig. 1.

## Results

### Analysis of annual publication trends

**Fig. 2:**
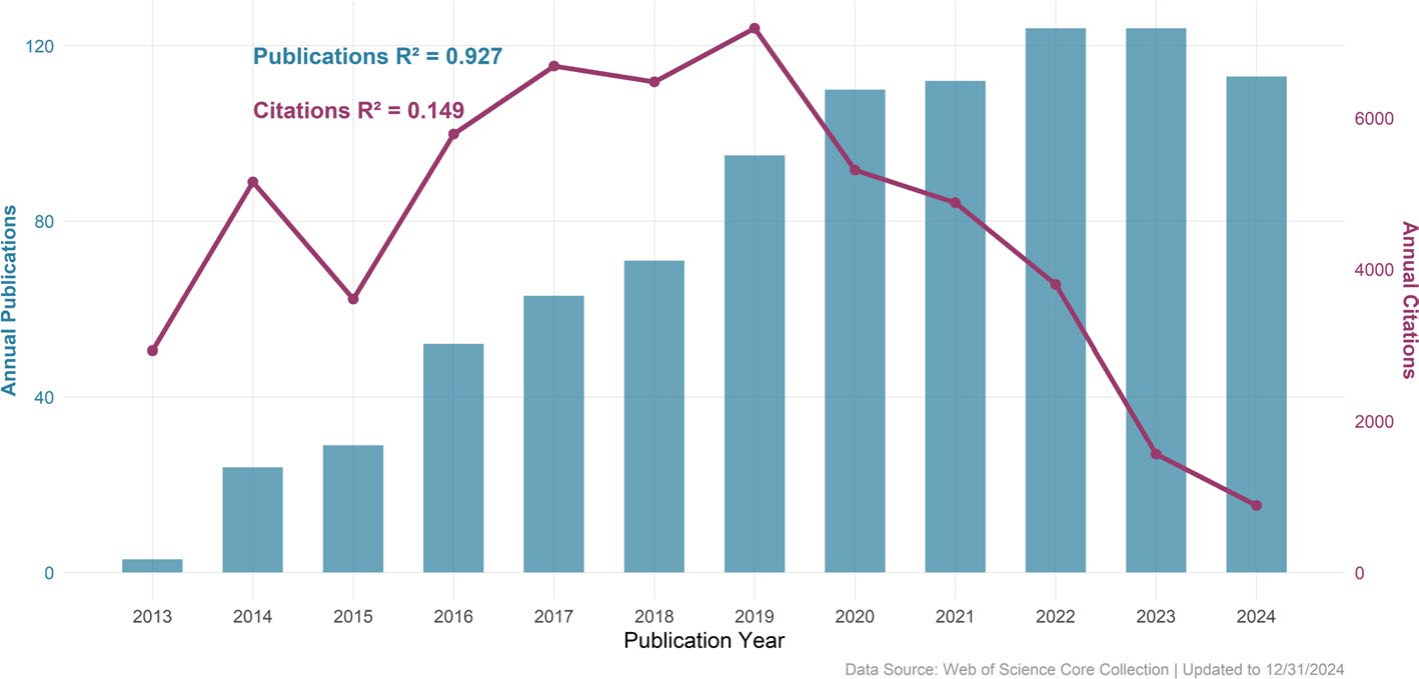
Annual publication and citation trends

A total of 928 publications from 2013 to December 31, 2024, were included in this study, comprising 736 articles (79.3%) and 192 reviews (20.7%). As illustrated in Fig. 2, annual publication volume in the field of SE-targeted cancer therapy have grown remarkably over the past decade. The trend reveals two pivotal milestones in 2019 and 2022, allowing us to categorize the evolution into three distinct phases as below. *Sprouting Stage (2013–2019)*: Publication volume surged from just 3 in 2013 to 95 in 2019, marking the emergence and early expansion of the field. *Stable Stage (2019–2022)*: Growth moderated during this period, with annual publication volume rising gradually from 95 to 124. *Peak Stage (2022–2024)*: Yearly output remained at a high level, stabilizing around 124 in 2023 and fluctuating within this range. Publication volume peaked at 124 in both 2022 and 2023. A strong correlation between publication numbers and year (R² = 0. 927) suggests that 2025 is likely to set a new record. In parallel, annual citations reached their highest point in 2019, with 7,184 citations, coinciding with the transition from the sprouting to the stable stage. However, overall citation counts did not show a significant correlation with time (R² = 0. 149), fluctuating around 4,000 annually in recent years. This may reflect the field’s maturation, as foundational discoveries from earlier years continue to anchor the citation landscape, while recent studies, though numerous, are still accumulating recognition.

In summary, both the volume of publications and their citation patterns confirm that super-enhancer-targeted cancer therapy remains in a vibrant phase of rapid development, attracting substantial and growing scholarly interest.

### Leading journals and intellectual influence

This study encompasses 928 publications on cancer therapy targeting SEs, disseminated across 295 scientific journals. As presented in Table 1, the top 20 journals, ranked by publication volume, are led by *Nature Communications* (*n* = 48), followed by *Cancer Research* (*n* = 30) and *Nucleic Acids Research* (*n* = 28). Notably, these 20 most productive journals, representing merely 6.78% of all journals, contributed 358 publications accounting for 38.58% of the total output. The preeminence of this research domain is further reflected in journal rankings. According to the Journal Citation Reports (JCR), 75. 6% of these journals are classified within the Q1 category, with 19.3% in Q2 and 5.1% in Q3, confirming the field’s established presence in high-impact, frontier science. In terms of Impact Factor (IF), *Nature* boasts the highest value (48.5), followed by *Cancer Cell* (44.5), *Cell* (42.5), *Cancer Discovery* (33.3), and *Nature Genetics* (29.0). The consistent prominence of such prestigious journals unequivocally highlights the exceptional academic value and cutting-edge nature of SE research in oncology.

**Fig. 3:**
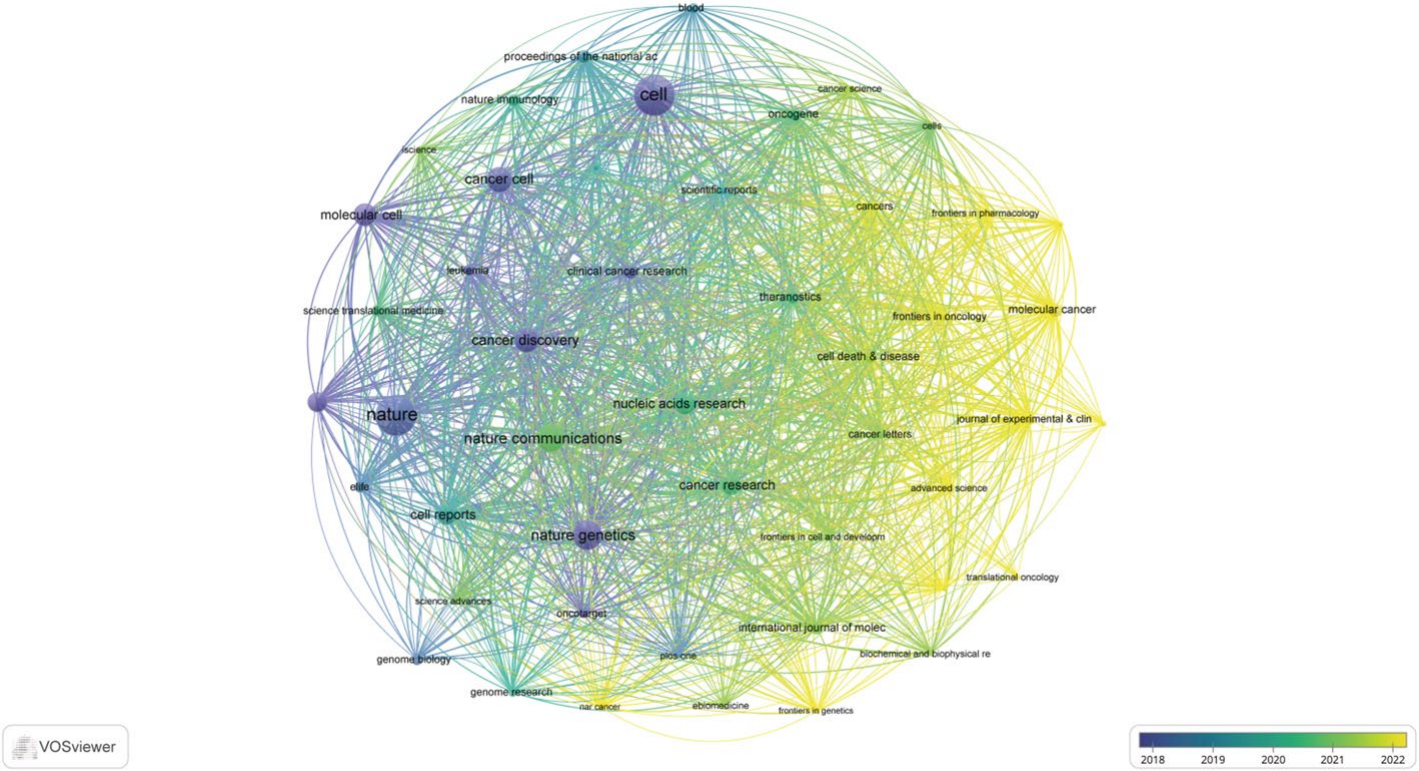
Overlay visualization map of productive journals. Circle size is proportional to citation count, and color represents the publication year on a gradient from blue (early) to yellow (recent)

Applying a publication threshold of five articles, we identified 48 highly productive journals for an in-depth citation analysis using VOSviewer (Fig. 3). Among these, Cell leads with 5,929 citations, followed closely by *Nature* (5,870) and *Nature Genetics* (2,966), underscoring their profound intellectual influence in the field.

**Table 1:** Top 20 journals for research of targeting SEs for cancer therapy

Rank	Journal	IF 2025	JCR quatile	Counts	Citations	Avg. citations	Avg. pub. year
1	Nature communications	15.7	Q1	48	2800	58	2020. 8
2	Cancer research	16.6	Q1	30	1314	44	2020. 4
3	Nucleic acids research	13.1	Q1	28	1633	58	2020. 2
4	Cell reports	6.9	Q1	22	1347	61	2019. 6
5	International journal of molecular sciences	4.9	Q1	22	577	26	2021. 1
6	Cell death & disease	9.6	Q1	21	512	24	2021. 4
7	Nature	48.5	Q1	20	5870	293	2018. 3
8	Cancers	4.4	Q2	18	267	15	2021. 7
9	Oncogene	7.3	Q1	17	628	37	2020. 4
10	Frontiers in oncology	3.3	Q2	15	266	18	2021. 8
11	Nature genetics	29.0	Q1	15	2966	198	2017. 8
12	Cancer letters	10.1	Q1	14	255	18	2021. 1
13	Cancer discovery	33.3	Q1	13	2014	155	2017. 8
14	Cell	42.5	Q1	13	5929	456	2017. 5
15	Cancer cell	44.5	Q1	11	2217	202	2018. 1
16	Proceedings of the national academy of sciences of the United States of America	9.1	Q1	11	602	55	2019. 2
17	Theranostics	13.3	Q1	11	591	54	2020. 3
18	Frontiers in cell and developmental biology	4.3	Q1	10	158	16	2021. 1
19	Scientific reports	3.9	Q1	10	242	24	2019. 4
20	Advanced science	14.1	Q1	9	187	21	2023. 3

### Contributions of countries and institutions

**Fig. 4:**
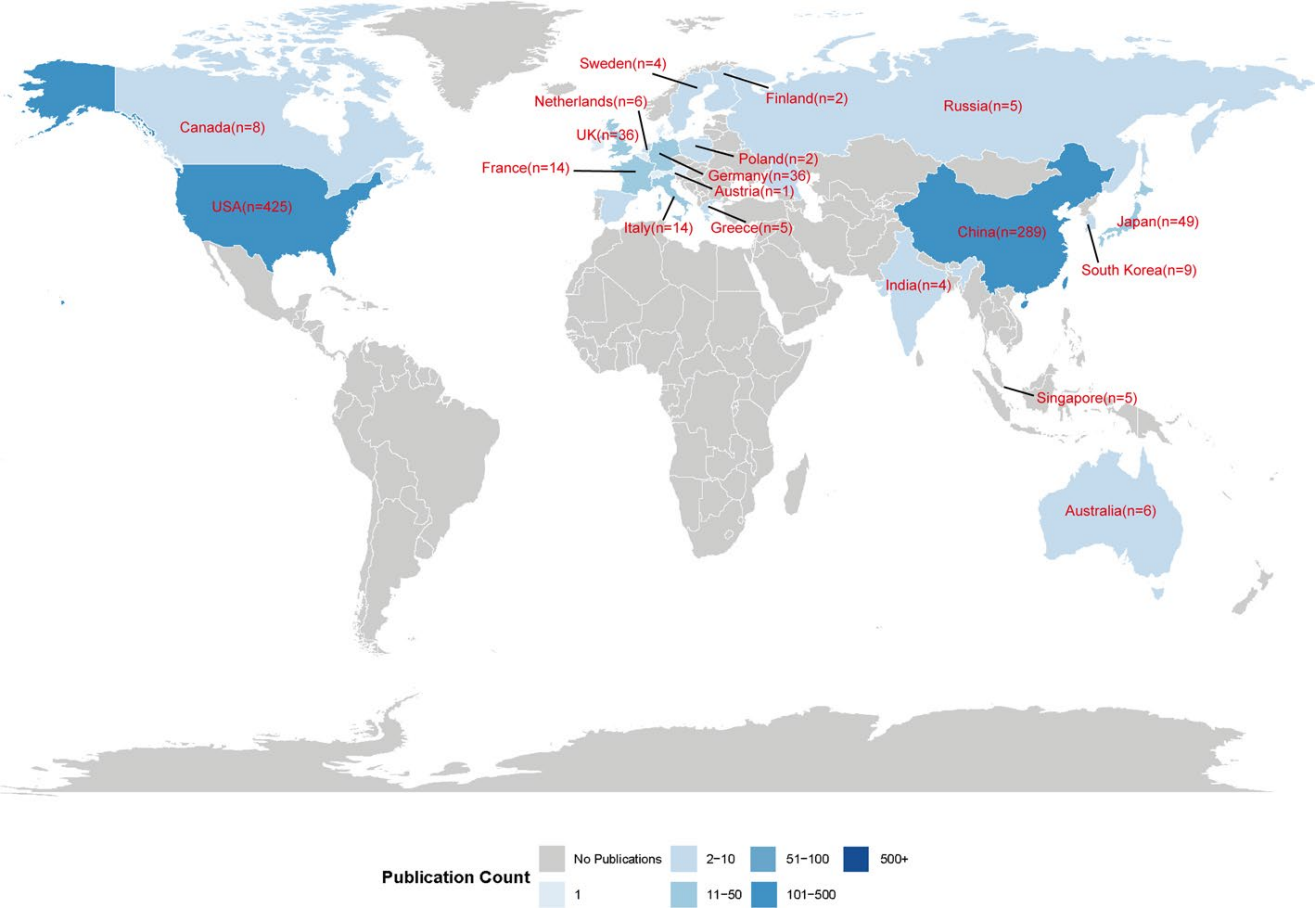
Global distribution of publications on targeting SEs for cancer therapy

**Fig. 5:**
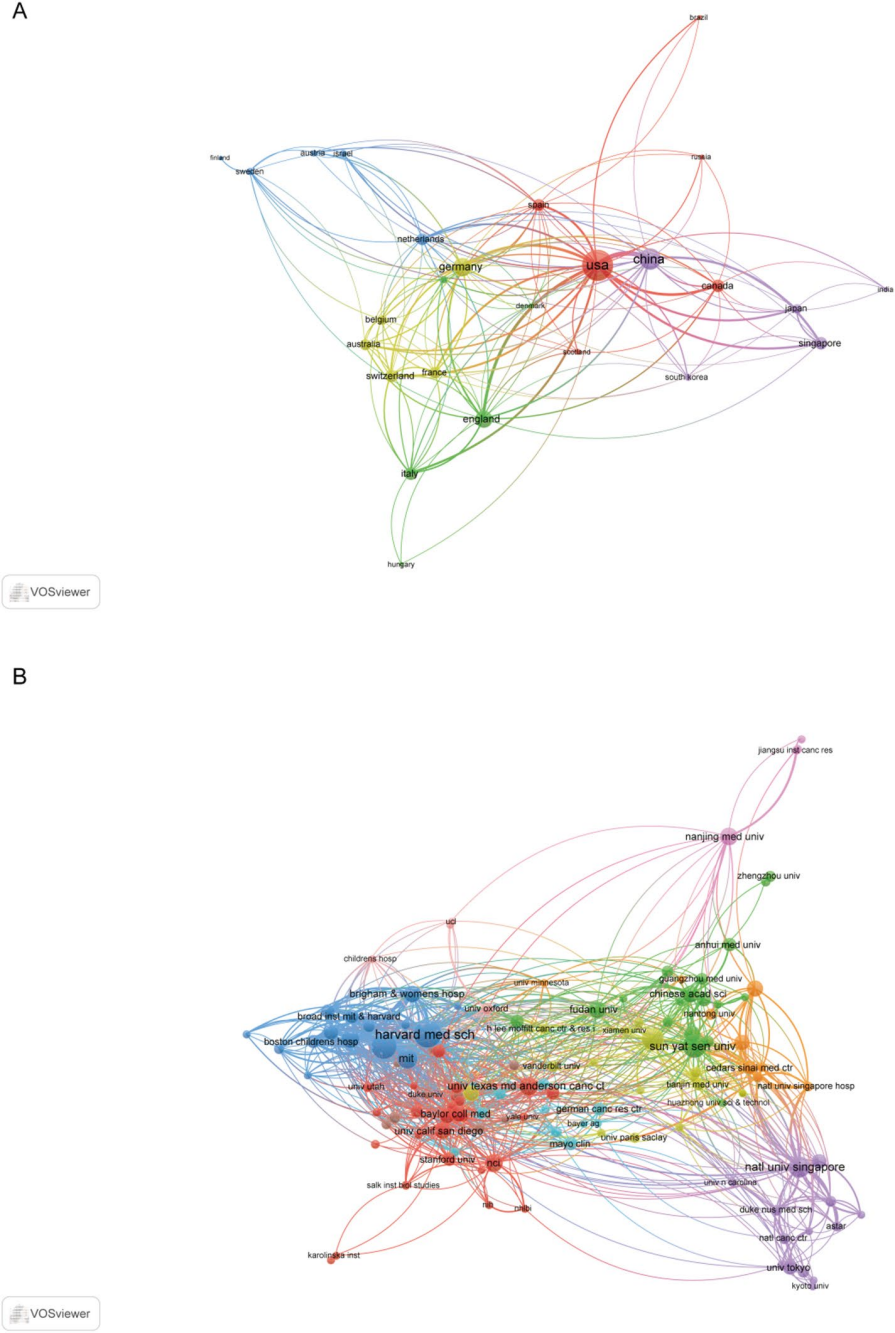
The visualization network of countries/regions and institutions. **A** The visualization network of countries/regions. **B** The network visualization of institutions. The size of the circles is proportional to the number of collaborations of countries/regions/institutions in the cooperation network. The thickness of the lines indicates the strength of the connection between countries/regions/institutions

Globally, 51 countries/regions have contributed to research on targeting SEs for cancer therapy (Fig. 4), reflecting the field’s considerable potential for future development. We have summarized the contributions of the top 20 most productive countries/regions based on publication volume, total citations, average citations per publication, and average publication year. As detailed in Table 2, the United States dominates the field with 425 publications, accounting for 45. 80% of the total output and significantly surpassing China (289 publications, 31.14%) and the Japan (49 publications, 5. 28%). In terms of scholarly impact, the United States also leads in total citations (38,024), followed by China (6,987) and Germany (2,504). Notably, Sweden achieved the highest average citations per publication (95), exceeding the United States (89.47) and Netherlands (78). Moreover, China has emerged as one of the most recent active contributors, with an average publication year of 2021.8. A collaboration network encompassing all 26 countries/regions was constructed (Fig. 5A). In this visualization, the size of each node corresponds to a country’s publication volume, while the thickness of the connecting lines represents the intensity of collaborative activity. The analysis reveals that the United States occupies a central position within the global network, demonstrating particularly strong collaborative ties with both China and the United Kingdom.

At the institutional level, 1,280 institutions have contributed to this field. Table 3 profiles the top 20 institutions by publication volume, detailing their geographical location, total citations, average citations per publication, and average publication year. We note that over half of the institutions (*n* = 11, 55. 0%) in this list are based in China, highlighting a distinct and prolific center of research activity. Harvard Medical School leads in both publication output (*n* = 42) and total citations (7,052), followed by Shanghai Jiao Tong University (*n* = 28) and Sun Yat-sen University (*n* = 21). Notably, the National Cancer Institute achieved the second-highest average citation count (106. 44), trailing only Harvard Medical School, despite a relatively modest publication volume (*n* = 9). Furthermore, Fujian Medical University emerged as the most recent active contributor among the top 20, with an average publication year of 2023. 6. By applying a minimum publication volume threshold of 12, we identified 125 high-yield institutions. A co-authorship analysis of these institutions using VOSviewer revealed a dominant collaborative network, which is structured into 10 distinct clusters (Fig. 5B). The largest cluster, represented in red, comprises 26 institutions. Within the entire network, Harvard Medical School cooperated with 43 high-yield institutions and Sun Yat-sen University cooperated with 21 high-yield institutions, underscoring their roles as pivotal hubs for scientific collaboration.

**Table 2:** Top 20 productive countries in targeting super-enhancers for cancer therapy research

Rank	Country	Counts	Total Citations	Avg. citations	Avg. pub. year
1	United States	425	38,024	89.47	2019.5
2	China	289	6987	24.18	2021.8
3	Japan	49	1100	22.45	2020.9
4	Germany	36	2504	69.56	2019.1
5	United Kingdom	28	1166	41.64	2019.2
6	France	14	830	59.29	2020.1
7	Italy	14	1023	73.07	2019.3
8	South Korea	9	192	21.33	2021.1
9	Canada	8	296	37	2020.9
10	Australia	6	241	40.17	2020.5
11	Netherlands	6	468	78	2017.3
12	Spain	6	141	23.5	2020.5
13	Greece	5	63	12.6	2021.2
14	Russia	5	42	8.4	2020.3
15	Singapore	5	164	32. 8	2020. 4
16	India	4	58	14. 5	2020. 2
17	Sweden	4	380	95	2018.8
18	Finland	2	42	21	2018.5
19	Poland	2	15	7.5	2021.5
20	Austria	1	71	71	2018.2

**Table 3:** Top 20 productive institutions in targeting super-enhancers for cancer therapy research

Rank	Institution	Location	Counts	Total Citations	Avg. citations	Avg. pub. year
1	Harvard Medical School	USA	42	7052	167.9	2017.7
2	Shanghai Jiao Tong University	China	28	885	31.61	2021.4
3	Sun-Yat Sen University	China	21	630	30	2021.7
4	Central South University	China	19	469	24.68	2021.8
5	National University Of Singapore	Singapore	19	810	42.63	2019.9
6	Nanjing Medical University	China	17	358	21.06	2022
7	Soochow University	China	13	195	15	2022.8
8	Columbia University	USA	11	826	75.09	2019.4
9	Anhui Medical University	China	10	151	15.1	2022.4
10	The University of Texas MD Anderson Cancer Center	USA	10	830	83	2018.8
11	Fudan University	China	9	130	14. 44	2020.9
12	National Cancer Institute	USA	9	958	106. 44	2019.4
13	Harbin Medical University	China	8	298	37. 25	2020.9
14	University of California, San Diego	USA	8	604	75. 5	2020.1
15	Wuhan University	China	8	201	25. 12	2020.5
16	Baylor College of Medicine	USA	7	477	68. 14	2020
17	Cedars-Sinai Medical Center	USA	7	455	65	2020.4
18	Fujian Medical University	China	7	34	4. 86	2023.6
19	Nagoya Univrsity	Japan	7	103	14. 71	2022.1
20	Nantong University	China	7	152	21. 71	2021.1

### Influential authors and co-cited authors

**Fig. 6:**
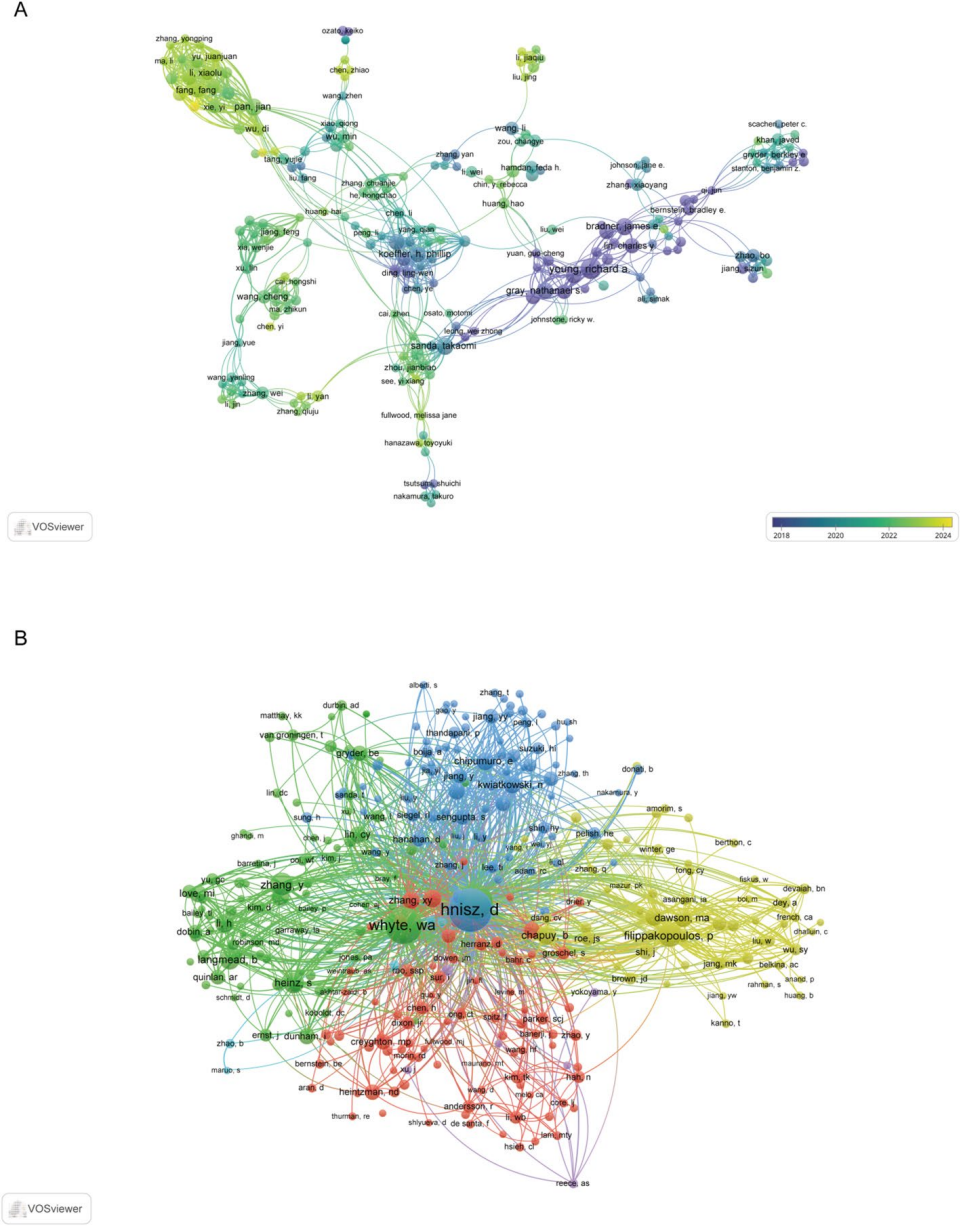
Author collaboration and co-citation networks in targeting SEs for cancer therapy **A** Author collaboration network and **B** Co-citation network

This analysis identified a total of 10,714 authors who have contributed to the field of super-enhancer-targeted cancer therapy. As summarized in Table 4, Young Richard A. leads the cohort with 22 publications and 8,629 citations, reflecting his foundational role in shaping this research domain. He is followed by Bradner James E. (17 publications, 6,571 citations), Abraham Brian J. (15 publications, 3,324 citations), and Gray Nathanael S. (15 publications, 3,181 citations), each having made substantial contributions to the literature. The collaborative network among authors is illustrated in Fig. 6A, which includes 220 researchers with a minimum of five publications each. In this visualization, node size corresponds to an author’s output, line thickness denotes collaboration intensity, and node color reflects temporal activity, shifting from purple (earlier engagement) to yellow (recent productivity). A central collaborative cluster includes Young Richard A., Bradner James E., Pan Jian, Li Xiaolu, and Sanda Takaomi, whose joint efforts have significantly advanced the understanding of SE mechanisms and their therapeutic targeting in oncology. It is also noteworthy that a number of recently active authors in the network are affiliated with Chinese institutions, underscoring China’s growing role in cutting-edge research within this dynamic field.

Furthermore, a co-citation analysis was conducted, identifying 29,084 co-cited authors in the literature. Table 4 ranks the top 20 most frequently referenced scholars. Three authors were co-cited on more than 500 times, with Hnisz D. (*n* = 807) being the most prominent, followed by Whyte W. A. (*n* = 576) and Loven J. (*n* = 501). To visualize intellectual linkages, a co-citation network was generated (Fig. 6B), including all authors meeting a minimum citation threshold of 20. The resulting network reveals significant intellectual clusters, exemplified by the strong co-citation ties between pairs such as Hnisz D. and Whyte W. A., as well as Filippakopoulos P. and Zhang Y., indicating their closely associated conceptual contributions to the field.

**Table 4:** Top 20 authors and co-cited authors in targeting super-enhancers for cancer therapy

Rank	Author	Counts	Citations	Co-cited author	Citations
1	Young Richard A.	22	8629	Hnisz D	807
2	Bradner James E.	17	6571	Whyte WA	576
3	Abraham Brian J.	15	3324	Lovén J	501
4	Gray Nathanael S.	15	3181	Filippakopoulos P	235
5	Pan Jian	14	248	Zhang Y	217
6	Lin Charles Y.	13	4557	Heinz S	179
7	Koeffler H. Phillip	12	863	Chapuy B	159
8	Lin Dechen	12	949	Langmead B	158
9	Sanda Takaomi	12	985	Li H	140
10	Zhang Tinghu	12	2974	Mansour MR	139
11	Fang Fang	11	90	Dawson MA	137
12	Li Xiaolu	10	75	Chipumuro E	136
13	Zhang Zimu	10	75	Delmore Je	134
14	Kwiatkowski Nicholas	9	2412	Zhang XY	129
15	Qi Jun	9	2110	Kwiatkowski N	127
16	Qu Jian	9	147	Subramanian A	125
17	Wang Jianwei	9	52	Bradner James E.	112
18	Wu Di	9	101	Lin Charles Y.	111
19	Brown Myles	8	1040	Pott S	107
20	Khan Javed	8	783	Sengupta S	107

### Analysis of references and co-citation network

**Fig. 7:**
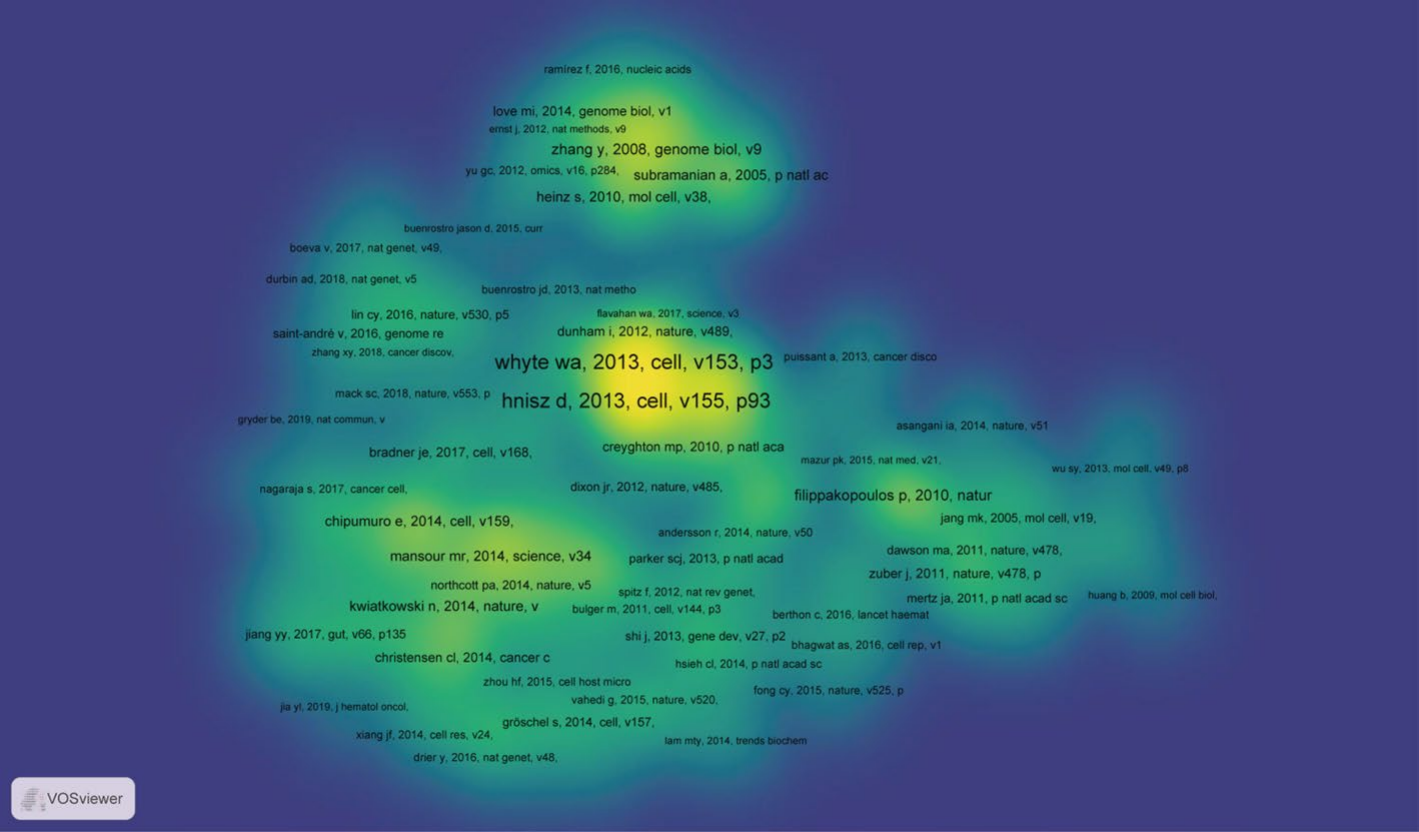
The co-citation density visualization of references cited within publications regarding targeting SEs and cancer research

Co-citation analysis examines intellectual linkages by identifying references that are cited together by subsequent publications. In the domain of SE-targeted cancer therapy, a total of 39,912 co-cited references were identified. As summarized in Supplementary Table 1, the most influential work is the 2013 *Cell* paper *Selective inhibition of cancer oncogenes by disruption of super-enhancers* by Loven J. et al., which had accumulated 2,285 co-citations by December 31, 2024. To delineate the foundational knowledge base, the 222 most frequently co-cited references (each meeting a threshold of 20 citations) were extracted for network analysis. Figure 7 presents a density visualization of these co-cited references, where brighter areas indicate higher citation frequency and intellectual centrality. This visualization highlights two seminal 2013 publications in Cell,*Master Transcription Factors and Mediator Establish Super-Enhancers at Key Cell Identity Genes* by Whyte W. A. et al., and *Super-enhancers in the control of cell identity and disease* by Hnisz D. et al. ,as the highest-density cores of the network, affirming their foundational role in shaping the field.

### Keyword co-occurrence and thematic analysis

**Fig. 8:**
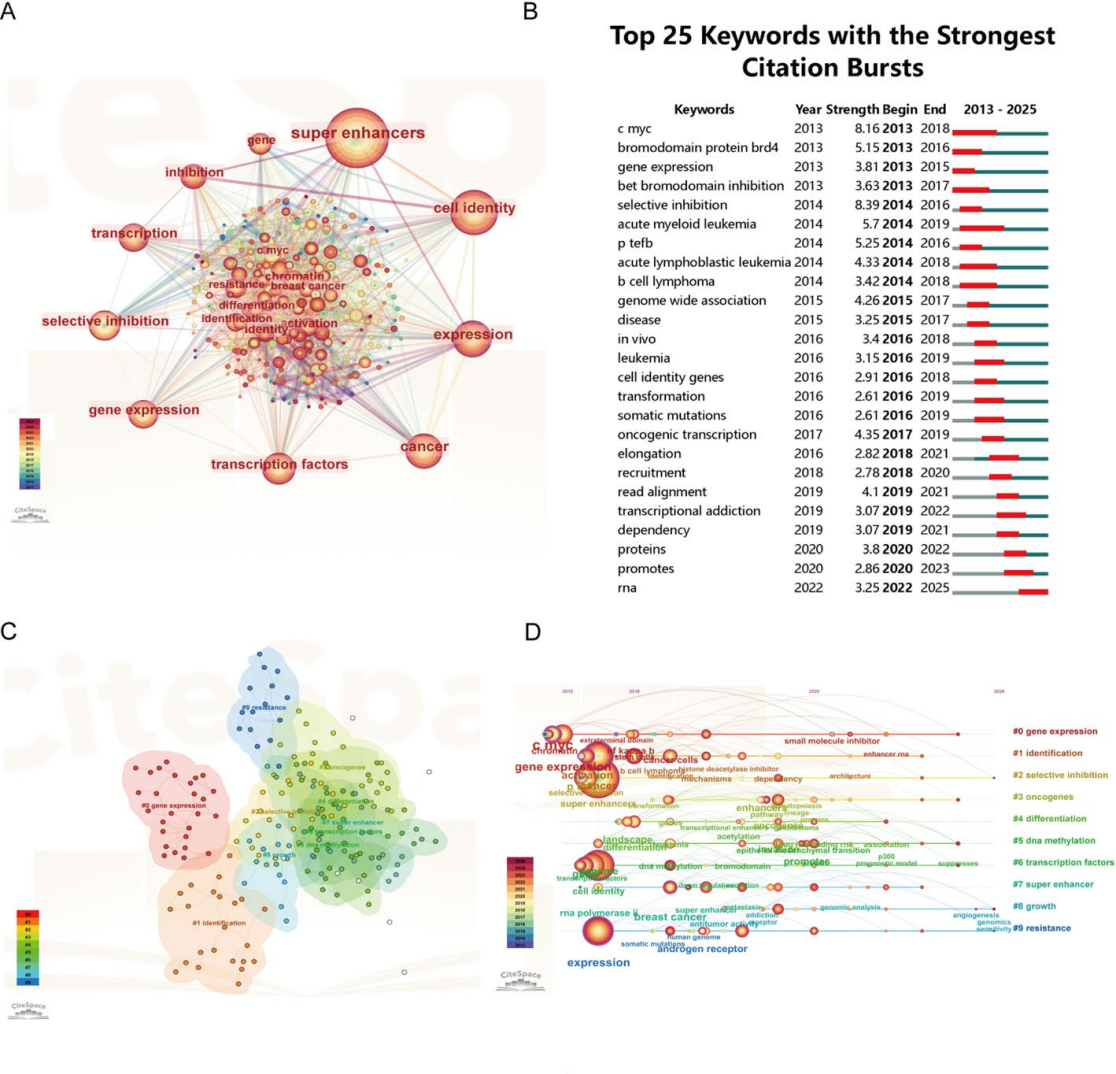
Keyword Co-occurrence and Thematic Analysis**A** Keyword co-occurence network **B** Top 25 keywords with the strongest citation bursts **C** Keyword clustering analysis **D** Temporal trends of keyword clusters

Keywords serve as concise summaries of research articles, and their analysis can reveal evolving hotspots and emerging trends in a scientific field. In this study, we performed a bibliometric keyword analysis on 928 publications focusing on SEs in cancer targeting therapy. A total of 3,307 keywords were identified, among which “super-enhancers” emerged as the most frequent term (n = 923), followed by “cell identity” (*n* = 478), “expression” (n = 436), and “cancer” (*n* = 330). In total, 10 keywords were mentioned more than 200 times. Besides those already noted, these included “transcription factors” (n = 292), “gene expression”(*n* = 262), “selective inhibition”(n = 257), “transcription”(*n* = 254), “inhibition” (n = 212), and “gene” (*n* = 201). To systematically outline the intellectual structure of this field, we conducted a keyword co-occurrence analysis using CiteSpace, applying a frequency threshold of 40 occurrences. This process identified 224 high-frequency keywords, which were visualized as a co-occurrence network (Fig. 8A). In the network, the size of each node corresponds to the frequency of the keyword, and the thickness of the connecting lines represents the strength of co-occurrence between terms. The analysis of keywords with strong citation bursts is summarized in Fig. 8B. Among the identified terms, “selective inhibition” exhibited the highest burst strength (8.39), followed by “c-myc” (8.16) and “acute myeloid leukemia” (5.7). These burst terms signify emerging research hotspots, with “selective inhibition” demonstrating sustained relevance, as its citation burst period extended until 2016.

Cluster analysis of keywords, conducted via CiteSpace, yielded 10 distinct clusters, each representing a unique research focus (Fig. 8C). The most prominent cluster, “#0 gene expression”, encompasses keywords such as “expression”, “transcription factors”, and “super enhancers”, underscoring the central role of transcriptional regulation in maintaining cell identity. Cluster “#1 identification” reflects methodological efforts in characterizing SE components and their functional associations. Cluster “#2 selective inhibition” highlights a key therapeutic strategy, focusing on small-molecule disruption of oncogenic SEs. Cluster “#3 oncogenes” emphasizes the relationship between SE activity and cancer-driving genes, particularly in the context of cancer initiation and maintenance. Cluster “#4 differentiation” links super-enhancer dynamics to cell lineage commitment and dedifferentiation processes in malignancy. Cluster “#5 DNA methylation” points to the interplay between epigenetic modifications and SE function. Cluster “#6 transcription factors” further elaborates the significance of master transcription factors in SE assembly and gene control. Cluster “#7 super enhancer” serves as the core thematic cluster, anchoring the general architecture and biological relevance of SEs across studies. Cluster “#8 growth” involves signaling and regulatory pathways influencing cell proliferation and survival via SE. Finally, Cluster “#9 resistance” suggests emerging interest in SE-mediated mechanisms of treatment resistance and adaptive cancer responses. Collectively, these clusters delineate a research landscape spanning from mechanistic insights into SE biology to translational investigations aimed at therapeutic targeting in cancer.

Meanwhile, based on the results of cluster analysis of the keyword, we conducted temporal analysis of keyword clusters to find dynamic changes in research focus. Figure 8D shows that gene expression-related study has initiated earliest in 2013. We can find that clusters like “identification” and “transcription factors” also emerged early and sustained until around 2023, indicating their fundamental role in the field. Conversely, clusters like “selective inhibition” and “resistance” exhibit emerging trend and potential future directions, demonstrating the expanding scope of SE-targeted cancer therapy research in addressing precise treatment and cancer resistance.

## Discussion

### Global research trend

Research on SEs in cancer targeted therapy has undergone remarkable growth over the past decade (Fig. 2). The period from 2013 to 2019 marked the sprouting stage of the field, during which annual publication volume surged from just 3 to 95,an increase of nearly thirty-fold, accompanied by a total of 37,812 citations. This phase not only witnessed rapid expansion in scholarly output but also established a conceptual and experimental foundation for SE research. Between 2019 and 2022, the field entered a stable growth stage. Annual publications rose gradually from 95 to 124, representing an increase of approximately 30%, while cumulative citations reached 51,800. During this phase, SEs gained broader recognition as a frontier oncological concept, with growing emphasis on their potential as therapeutic targets owing to their role in amplified oncogene transcription. Since 2022, the field has progressed into a peak stage, with annual publication volume stabilizing around 124. This plateau suggests a maturation of theoretical frameworks and a shift in research focus from fundamental mechanisms toward clinical translation. The increasing use of gene-editing technologies and specific small-molecule inhibitors has further strengthened the integration of SE insights into targeted cancer treatment strategies [24, 32]. This trend reflects a growing enthusiasm for interdisciplinary collaboration and efforts to overcome barriers in SE-directed therapy. The consistent expansion of research in this domain provides a solid basis for advanced molecular studies, clinical technology development and the systematic translation of basic findings into precision oncology contributing significantly to the advancement of personalized cancer care.

The study reveals distinct yet complementary roles among leading countries in SE cancer targeted therapy research. The United States and China have emerged as the two central contributors, collectively accounting for 76. 3% of total publications. The United States leads in both output and influence, with 425 publications and 38,024 total citations. China follows with 289 publications and 6,987 citations, yet demonstrates more recent activity, with average publication year of 2021. 8 surpasses that of the U.S. by nearly two years (Table 2). This indicates that while the U.S. has established a foundational academic influence, China is increasingly active at the research frontier. This divergence can be attributed to several factors. The United States benefits from longstanding scientific infrastructure, including world-renowned institutions and pioneering researchers epitomized by Young Richard A. ’s seminal work at the Whitehead Institute [15, 16, 19]. In contrast, China’s rapid ascent has been propelled by sustained governmental funding and strategic policy support, fostering the growth of leading academic centers such as Shanghai Jiao Tong University, Sun Yat-sen University, and Central South University. U.S. institutions, represented largely by purple and blue nodes, have historically shaped the theoretical and mechanistic foundations of the field(Supplementary Fig. 1). Harvard Medical School, for instance, ranks first in both publication volume and total citations. Its scholarly impact exceeding the combined citations of the second through thirteenth ranked institutions. (Table 3). The institutional collaboration network further illustrates these trends (Fig. 5B). Meanwhile, Chinese institutions such as Zhengzhou University and Southeast University appear in yellow tones, signaling their recent and growing contributions. Beyond the U.S. and China, other Asian institutions are gaining prominence. The National University of Singapore and Japan’s Nagoya University also represented by yellow nodes, highlighting a broader geographic shift in research activity toward East and Southeast Asia(Supplementary Fig. 1). Among them, the National University of Singapore exhibits particularly strong cross-regional ties with both China and the U.S. As China continues to enhance its productivity and citation impact, it is poised to play an increasingly central role in global collaborations. Together, these dynamics suggest that the future of SE-targeted cancer research will be increasingly shaped by international and interdisciplinary partnerships, with Asian institutions taking a leading role in translational and innovative studies.

### Advanced landscape and cutting-edge field

####  Thematic structure and temporal evolution of research fronts

Keyword co-occurrence analysis offers valuable insights into the intellectual structure and thematic evolution of SE-targeted cancer therapy research. In this study, a total of 3,307 keywords were identified from 928 publications. High-frequency terms such as “super-enhancer”, “cell identity”, “expression”, “cancer”, “transcription factors”, and “selective inhibition” delineate the core themes of the field (Fig. 8A). Cluster analysis performed with CiteSpace grouped co-occurring keywords into 10 distinct thematic clusters (Fig. 8C), providing an objective framework of the research landscape.

A temporal analysis of these clusters reveals a clear progression in research focus (Fig. 8D). The earliest and most central cluster, “#0 gene expression”, emerged in 2013, coinciding with the foundational conceptualization of SEs. This was followed closely by clusters such as “#1 identification” and “#6 transcription factors”, which sustained attention until around 2023, indicating their enduring role in defining SE biology. In contrast, clusters like “#2 selective inhibition” and “#9 resistance” gained prominence in more recent years, highlighting a shift toward translational and therapeutic challenges.Meanwhile, emerging terms including “epigenetic regulation”, “resistance” and “bromodomain inhibitor” reflect the ongoing expansion of the research landscape toward novel mechanistic and therapeutic dimensions.

At the level of specific molecular targets and pathways, the keyword analysis further delineates publication trends. Terms directly naming therapeutic targets (e.g.”BRD4”, “CDK7”, “CDK9”) and drug classes (e.g.”BET inhibitors”, “bromodomain inhibitor”) are predominantly concentrated within the “selective inhibition” cluster and show a marked increase in co-occurrence frequency from 2020 onward. Meanwhile, keywords associated with resistance mechanisms (e.g., “somatic mutations”, “drug efflux”) are largely contained within the ”resistance” cluster, with their strongest citation bursts occurring in the period 2020–2024.

#### Biological interpretation and therapeutic significance of key themes

Using complementary bibliometric techniques, we quantitatively mapped the evolving research fronts and emerging trends in SE-based translational science, collectively outlining the current research framework and highlighting areas with significant potential for future investigation. Building upon the objective bibliometric patterns, we next interpret the biological and clinical significance of the dominant research themes.

The enduring focus on “gene expression” represents one of the most central and long-standing research themes.Gene expression, governed by the central dogma of molecular biology, entails a multi-step process involving RNA transcription and protein translation [33], tightly regulated by DNA regulatory elements such as enhancers. It establishes a root from which numerous subsequent research branches have sprouted and underscores the foundational principle that SEs function as potent cis-regulatory modules in SE-driven cancer biology. Their ability to recruit master transcription factors, coactivators like the Mediator complex and BRD4, and RNA Polymerase II drives the high-level transcription of genes critical for cell identity and oncogenesis [15, 16, 33]. The frequent co-occurrence of “BRD4” within this cluster is not merely a bibliometric artifact.It reflects the protein’s established biological role as a key reader and facilitator of SE-driven transcription [34, 35]. This mechanistic understanding directly explains why BRD4 emerged as a primary therapeutic target.Recent studies continue to validate its relevance, demonstrating that BRD4 inhibition suppresses tumor growth in diverse malignancies, including glioblastoma [36], hepatocellular carcinoma [37], and T-cell leukemia [38].

The rise of the “selective inhibition” cluster reflects a key translational focus in SE research which is the development of therapeutic strategies that specifically disrupt SE-driven oncogenic transcription. It is closely linked to the foundational “#0 gene expression” cluster and marks the field’s transition from mechanistic discovery to therapeutic innovation. The prominence of keywords like “BET inhibitors” corresponds to the first wave of pharmacologic strategies aimed at disrupting the SE machinery. BET inhibitors represent a promising class of anticancer agents that function by competitively binding to acetyl-lysine recognition sites, thereby disrupting the interaction between bromodomain-containing proteins like BRD4 and acetylated histones at SEs. This interference impairs SE activity, reduces expression of oncogenes such as MYC, and ultimately inhibits tumor growth [39].Among these agents, JQ1 is a well-characterized small-molecule inhibitor that binds directly to the bromodomain of BRD4, preventing its association with acetylated chromatin [40]. By displacing BRD4 from SE regions marked by H3K27ac, JQ1 disrupts the enhancer-promoter interactions necessary for robust transcription of oncogenes [19]. Because SE-driven transcription is highly sensitive to the availability of key transcriptional regulators, JQ1 selectively targets malignant cells while largely sparing normal tissues [15, 19].Further refinement of this approach has led to the development of proteolysis-targeting chimeras (PROTACs) such as dBET1, which not only block BRD4 binding but also induce its degradation, offering enhanced efficacy and specificity [41, 42]. This trend in the literature mirrors the clinical and preclinical evolution from pan-BET inhibitors toward more selective compounds and novel degradation modalities, which aim to improve efficacy and reduce toxicity [39–42]. Our analysis also shows that recent keyword bursts (2021–2024) are associated with next-generation agents, such as “OTX015”. In addition to BET inhibitors, other SE-targeting agents, including CDK7 and CDK9 inhibitors, have shown promise in abating oncogenic transcription [43].Many of these compounds, including I-BET762, OTX015, and CPI0610, have now entered clinical evaluation [44–46].Collectively, these inhibitors exemplify a rational therapeutic strategy, which selectively incapacitating cancer cells by disrupting oncogene transcription driven by SEs. A summary of their mechanisms and status is provided in Supplementary Table 2.

The crystallization of “resistance” as a distinct cluster highlights a critical translational frontier. The bibliometric emergence of this cluster does not simply indicate that resistance is a frequently used term.Rather, it signals the research community’s concerted effort to understand a major clinical barrier. Keywords such as “somatic mutations”, “tumor suppressor resistance”, “activating mutations”, outline the current research landscape concerning therapeutic resistance in this field. Growing evidence indicates that SEs contribute to cancer progression and drug resistance by driving the aberrant expression of oncogenes and resistance-related genes [47, 48]. Similar to conventional chemotherapy, resistance to SE-directed therapy arises through diverse mechanisms, including adaptation to oxidative stress, dysregulation of lipid metabolism, activation of complement signaling pathways, phenotypic plasticity, target gene mutations and overexpression of multidrug resistance genes [49–54]. These adaptive responses collectively undermine the effectiveness of targeted treatment strategies.Recent studies have begun to elucidate the specific roles of SEs in mediating therapy resistance. For example, in certain cancers, SEs have been shown to directly upregulate genes associated with drug efflux and survival [26]. Additionally, transcriptional kinases such as CDK9 and CDK12, which phosphorylate RNA polymerase II and facilitate SE-driven transcription, have emerged as potential targets for overcoming resistance. Inhibition of these kinases can disrupt oncogenic transcriptional programs and impair adaptive resistance to agents such as SMO inhibitors [55].Notably, while chemoresistance remains a common challenge in conventional chemotherapy, emerging strategies that directly target SE components or their co-regulators show promise in resensitizing tumors to treatment and improving clinical outcomes [56]. These mechanistic insights not only deepen our understanding of SE-mediated resistance but also reveal novel therapeutic vulnerabilities, offering a roadmap for future translational research in this evolving area.

Critically, recent evidence underscores that the adaptive transcriptional plasticity of SEs themselves is a primary driver of resistance, particularly to BET inhibitors. Prolonged exposure to BET inhibitors like JQ1 can trigger a compensatory rewiring of SE landscapes, where tumors circumvent BRD4 dependency by forming de novo SEs at alternative genomic loci to sustain oncogene expression [57, 58]. This SE remodeling represents a non-genetic, transcriptionally driven escape mechanism that directly links SE biology to treatment failure. Furthermore, the up-regulation of drug efflux pumps and metabolic reprogramming are often orchestrated by these same dynamically altered SEs [26, 47, 49]. Therefore, effectively countering SE-mediated resistance may require strategies that not only inhibit SE activity but also prevent or disrupt the epigenetic plasticity that allows SEs to adapt, highlighting the potential of combination therapies targeting both the SE machinery and the chromatin regulators that facilitate its remodeling.

#### Therapeutic evolution: from pan-BET inhibitors to selective agents and protein degraders

Our bibliometric keyword trends, notably the shift from broad terms like “BET inhibitors” to more specific descriptors, directly mirror a profound evolution in the drug discovery paradigm for SE-targeted therapy. This progression is driven by the dual imperative to enhance therapeutic efficacy and overcome adaptive resistance, as outlined in the previous section.The foundational work in this field leveraged first-generation pan-BET inhibitors such as JQ1 and I-BET762. These compounds competitively occupy the acetyl-lysine binding pockets of both BD1 and BD2 bromodomains within BRD4 and related proteins, globally disrupting their chromatin localization and SE-driven transcription. While proving the therapeutic concept, their broad-spectrum activity is associated with dose-limiting toxicities (e.g., thrombocytopenia, gastrointestinal effects) and potential off-target effects, limiting their clinical therapeutic indices.

In response, the field has undergone a decisive shift toward domain-selective inhibitors, a trend clearly reflected in recent patent analyses [59]. BD1-selective compounds (e.g., GSK789, ABBV-744) can achieve more than 100-fold selectivity for BD1 over BD2, while BD2-selective agents like RVX-208 (apabetalone) exhibit the converse preference (170-fold selectivity). This selectivity is structurally grounded in subtle differences in the amino acid residues lining the acetyl-lysine binding pockets of the two domains [59–61]. The clinical rationale is compelling that BD1 inhibition appears to be the primary driver of antitumor efficacy in many malignancies, whereas BD2 inhibition may play a more dominant role in modulating inflammatory gene programs. Therefore, BD1-selective agents aim to retain anticancer activity while mitigating toxicity, potentially expanding the therapeutic window. Furthermore, BD2-selective agents like apabetalone (currently in Phase 3 trials for cardiovascular complications of diabetes) illustrate how this selectivity can reposition SE-targeting strategies beyond oncology, into areas like inflammation and fibrosis [61].

Despite improvements in selectivity, traditional inhibitors are inherently reversible and susceptible to compensatory mechanisms, such as the upregulation of target protein expression or SE remodeling. This limitation has catalyzed the rapid development of targeted protein degradation strategies, chiefly Proteolysis-Targeting Chimeras (PROTACs) and related molecular glues. PROTACs (e.g., dBET1, ARV-771, MZ1) are heterobifunctional molecules that simultaneously bind BRD4 and an E3 ubiquitin ligase (e.g., VHL or CRBN), inducing polyubiquitination and proteasomal degradation of the target [62, 63]. This offers several key advantages over inhibition, including sustained pharmacological effect persisting beyond drug clearance, catalytic mode of action requiring sub-stoichiometric doses, potential to degrade “undruggable” proteins that lack conventional binding pockets, and potential to overcome resistance driven by compensatory protein overexpression or conformational changes that affect inhibitor binding.An even more recent innovation is the class of intramolecular bivalent glues (IBGs), such as IBG1. These monovalent molecules induce targeted degradation by simultaneously engaging both BD1 and BD2 domains of BRD4, stabilizing a conformation that recruits an E3 ligase for degradation via a unique, glue-like mechanism [64]. This represents a sophisticated evolution in degrader design with potential for improved physicochemical properties. Critically, both PROTACs and IBGs directly address the core resistance mechanism of SE plasticity. By removing the BRD4 protein entirely, rather than temporarily blocking its function, they offer a more durable means to collapse the SE complex and prevent the transcriptional rewiring that allows tumors to escape traditional inhibitors.

The trajectory captured in our analysis that from pan-inhibitors to selective agents and now to degraders defines the current frontier of SE-targeted drug development and charts its future course. This evolution is poised to advance along several interconnected paths, including the refinement of highly optimized, selective inhibitors with best-in-class profiles for specific malignancies or even non-oncologic indications, the design of dual-target degraders or hybrid molecules that co-target BRD4 alongside other core SE components (e.g., transcriptional kinases like CDK9) or critical resistance pathways, the engineering of next-generation degraders with enhanced oral bioavailability, tissue specificity, and minimized off-target ligase engagement. Notably, the strategic development of rational combination therapies that pair SE-directed agents with immunotherapy, chemotherapy, or other epigenetic modulators to preempt or overcome resistance. This continuous innovation cycle underscores that targeting SEs is not a static therapeutic strategy but a dynamically evolving field, where pharmacologic advances are inextricably linked to a deepening understanding of transcriptional biology and tumor adaptation.

#### Synthesis and future directions

The sequential emergence of these themes from foundational biology (“gene expression”) to therapeutic intervention (“selective inhibition”) and finally to clinical barrier (“resistance”) maps a coherent translational trajectory for SE-targeted cancer therapy.The bibliometric trends indicate two primary, interconnected future directions. First, the evolution within the “selective inhibition” cluster underscores the imperative to advance the pharmacological arsenal. This will likely involve a shift from broad-spectrum agents toward more selective compounds (e.g., BD1- or BD2-specific BET inhibitors) and novel modalities like PROTACs and molecular glues, aimed at enhancing efficacy and overcoming the resistance mechanisms outlined in cluster #9. Second, to effectively counter the adaptive resilience captured by the “resistance” cluster, future research must prioritize understanding and targeting the dynamic rewiring of SEs themselves. This includes elucidating how SEs drive non-genetic resistance pathways (e.g.metabolic reprogramming) and developing rational combination therapies that simultaneously disrupt SE activity and block emergent survival signals.

### Enhancer rnas: promising frontiers of SE in cancer therapeutic target

This study further illuminates enhancer RNAs as promising frontiers in targeting SEs for cancer therapy. Burst keyword analysis reveals “selective inhibition” as the term with the highest citation burst intensity in SE-related research, maintaining prominence until 2016, followed by “c-Myc” and “acute myeloid leukemia”. Notably, while “RNA” exhibits more moderate burst strength, its emergence during the 2022–2025 period marks it as the most recent keyword among the top 25 with strong citation bursts (Fig. 8B). This conceptual evolution is clearly reflected in the temporal trajectory of keyword clusters. Specifically, within the “#0 gene expression” cluster (Fig. 8D), the keyword “enhancer RNA” has been tracked as a rapidly emerging focus, signaling its growing importance and potential value for advancing precision medicine and countering mechanisms of tumor resistance. This pattern signals a conceptual evolution in the field, shifting attention beyond protein-coding targets toward the non-coding RNA outputs of SEs. Historically, SE research has emphasized transcriptional regulation of protein-coding genes. However, growing evidence indicates that SEs are themselves actively transcribed, yielding non-coding RNAs referred to as enhancer RNAs (eRNAs), which are now understood to be functional regulators rather than mere transcriptional byproducts [65, 66].

Enhancer RNAs (eRNAs) are a class of long non-coding RNAs transcribed from active enhancers, including SEs [67]. Emerging evidence indicates that eRNAs are not mere transcriptional byproducts but functional regulators that actively mediate SE activity through multiple mechanisms.First, eRNAs facilitate the physical proximity between enhancers and promoters by participating in or stabilizing chromatin looping, a prerequisite for robust gene activation. Second, they act as molecular scaffolds that recruit and retain key transcriptional co-activators, such as BRD4 and components of the Mediator complex at SE regions, thereby amplifying transcriptional output. Third, eRNAs contribute to the stabilization of the transcription pre-initiation and elongation machinery at target gene loci, ensuring sustained oncogene expression [68].Beyond these direct roles, the mapping and quantification of eRNA loci have provided a functional readout of SE activity that complements traditional chromatin marks [69]. This approach enhances our ability to link SEs to phenotypic traits and disease mechanisms, opening new avenues to investigate SE function in development and pathology.Collectively, these mechanisms establish eRNAs as central players in SE-driven transcriptional programs. This paradigm also reveals a therapeutic opportunity, while directly inhibiting SE-bound oncoproteins or editing SE loci remains technically challenging, targeting upstream eRNAs offers a novel strategy to disrupt oncogenic transcription at its source, with potential advantages in specificity and reduced off-target effects.

Recent studies have established the central role of enhancer RNAs (eRNAs) in mediating enhancer-promoter interactions and driving tumor oncogenesis and progression, positioning them as promising therapeutic targets in several aggressive cancers, including prostate, breast, renal clear cell, and pancreatic carcinomas [70]. Meanwhile, these frontier studies establish a solid theoretical foundation for developing eRNA-targeting strategies.In hormone-driven malignancies such as prostate and breast cancer, eRNAs are intricately involved in androgen receptor (AR)- and estrogen receptor (ER)-mediated transcriptional programs [71]. A notable example is the lactotransferrin-eRNA (LTFe) in prostate cancer, which is frequently downregulated and exerts a tumor-suppressive function. LTFe promotes the transcription of its host gene LTF by interacting with HNRNPF and facilitating chromatin looping. Crucially, the LTFe-LTF axis enhances ferroptosis by modulating iron transport, whereas AR signaling disrupts this axis and confers ferroptosis resistance, highlighting LTFe as a potential epigenetic therapeutic target whose activity could be restored via eRNA-directed strategies [65].In breast and pancreatic cancers, eRNAs also contribute to malignancy through epitranscriptomic mechanisms. For instance, certain eRNAs promote tumor progression via N6-methyladenosine (m6A) modification, which stabilizes eRNA transcripts and amplifies their oncogenic functions [72, 73]. This regulatory layer presents another druggable node, that is, small molecules or oligonucleotides that interfere with m6A deposition or recognition could selectively disrupt eRNA-dependent oncogenic circuits.Beyond these mechanisms, recently identified eRNAs such as AC003092.1 in renal clear cell carcinoma and MYC enhancer RNA in pancreatic cancer are associated with poor prognosis, further underscoring their clinical relevance and potential as candidate therapeutic targets [74, 75].In summary, eRNAs represent a therapeutically actionable class of non-coding RNAs. Emerging targeting strategies include antisense oligonucleotides (ASOs) [76, 77] designed to degrade or sequester specific oncogenic eRNAs such as AC003092.1 or MYC eRNA mentioned above, small-molecule inhibitors that disrupt eRNA-protein interactions like LTFe-HNRNPF or modulate epitranscriptomic regulators (e.g.m6A writers/readers) [78], and chromatin-targeting approaches that restore tumor-suppressive eRNA loops(e.g. LTFe-LTF axis) in hormone-resistant cancers above.These strategies may enable more precise transcriptional interference with reduced off-target risks.

Based on the preceding analysis, eRNAs emerge as promising druggable nodes due to a combination of unique functional and expression characteristics.Beyond their direct mechanistic roles in mediating SE activity, such as through chromatin looping, co-activator recruitment, and transcriptional complex stabilization, eRNAs are integrated into broader epigenetic and signaling networks. They participate in key regulatory interactions, such as binding to specific proteins and can undergo post-transcriptional modifications (e.g.m6A methylation) that fine-tune their stability and function.Critically, eRNAs exhibit highly tissue-specific and cancer-type-specific expression patterns. Systematic profiling using resources such as The Cancer Genome Atlas (TCGA) and the Genotype-Tissue Expression (GTEx) project has mapped eRNA landscapes across normal and malignant tissues, revealing that many eRNAs are dysregulated in particular cancers [79, 80]. This specificity reduces the likelihood of on-target toxicity in non-diseased tissues and enhances their potential as selective therapeutic targets.Moreover, their disease-restricted expression positions eRNAs as attractive biomarkers for diagnosis and therapeutic stratification. Several studies have already identified individual eRNAs whose levels correlate with prognosis or treatment response, supporting their translational relevance [81–84].In summary, eRNAs represent druggable nodes not only because they occupy functionally essential positions within SE-driven oncogenic circuits but also because they offer favored pharmacological properties: molecular accessibility, functional indispensability in cancer cells, and expression patterns that enable precise targeting within a precision oncology framework.

### limitations and future directions

While bibliometric analysis offers a powerful approach for mapping scientific landscapes, this study is subject to several inherent limitations. Firstly, the data source was restricted to the Web of Science Core Collection(WoSCC) database [85]. Although WoSCC provides high-quality, curated records, it may not comprehensively capture all relevant literature indexed in other databases such as Scopus, PubMed, or Google Scholar. Future studies could adopt a multi-database search strategy and employ data fusion techniques to create a more inclusive and representative dataset. Secondly, despite efforts to standardize terminology through manual keyword annotation, challenges persist due to conceptual overlap, evolving jargon, and disciplinary differences in vocabulary. These factors can affect the precision of keyword-based clustering and trend detection. Subsequent research could employ natural language processing (NLP) models trained on domain-specific corpora to further refine semantic analysis and improve thematic classification. A third limitation stems from citation lag. Recently published high-quality works have not had sufficient time to accumulate citations, which may lead to an underestimation of their eventual academic impact. To mitigate this, future analyses could complement citation counts with alternative metrics, such as Altmetric attention scores or early citation rates, to better identify emerging but influential studies. Finally, the methodological scope of this study is inherently quantitative. While tools like VOSviewer and CiteSpace excel at revealing macroscopic trends, collaboration patterns, and conceptual networks, they do not support qualitative assessment of research content such as experimental rigor, methodological innovation, or clinical validity. Follow-up research should integrate systematic content review or expert surveys to contextualize bibliometric findings and appraise the substantive contributions of key publications.

Notwithstanding these limitations, the trends identified in this study, including the growing emphasis on gene expression regulation, selective inhibition, drug resistance, and enhancer RNAs, provide valuable insights for the field. By delineating these evolving frontiers, our findings help prioritize research avenues with strong clinical translation potential, stimulate interdisciplinary partnerships, and inform the development of next-generation cancer therapies targeting SE dysfunction.

## Conclusion

This bibliometric analysis synthesizes a decade of global research on targeting super-enhancers for cancer therapy, charting its trajectory from conceptual emergence to a field poised at a pivotal juncture. We reveal a dynamic landscape where China leads in quantitative output and the United States in scholarly impact, driven by key institutions and foundational investigators. The evolution of research fronts, from gene expression and selective inhibition to the pressing challenge of drug resistance and the nascent promise of enhancer RNA, maps a clear shift toward translational applications. These findings not only consolidate the current intellectual architecture but also provide a strategic compass for future endeavors, emphasizing the need to translate mechanistic insights into clinical strategies that overcome therapeutic resistance and advance precision oncology.

## Supplementary Information

Below is the link to the electronic supplementary material.

Supplementary Material 1

## Data Availability

The original contributions presented in the study are included in the article/supplementary material, further inquiries can be directed to the corresponding author.

## Abbreviations

SE: Super-enhancer
WoSCC: Web of science core collection
ncRNAs: Non-coding RNAs
BRDs: Bromodomain-containing proteins
eRNAs: Enhancer RNAs
