# Supplementary material for: The research landscape and future of targeting super-enhancers for cancer therapy: a bibliometric analysis
Source: Discov Oncol. 2026 Jan 28;17:351. doi: 10.1007/s12672-026-04471-w (PMC12923725; doi:10.1007/s12672-026-04471-w)
Supplement: Supplementary file 1 — Supplementary Material 1 [file 12672_2026_4471_MOESM1_ESM.docx]

Supplementary Material

# Supplementary Figures and Tables

## Supplementary Figures


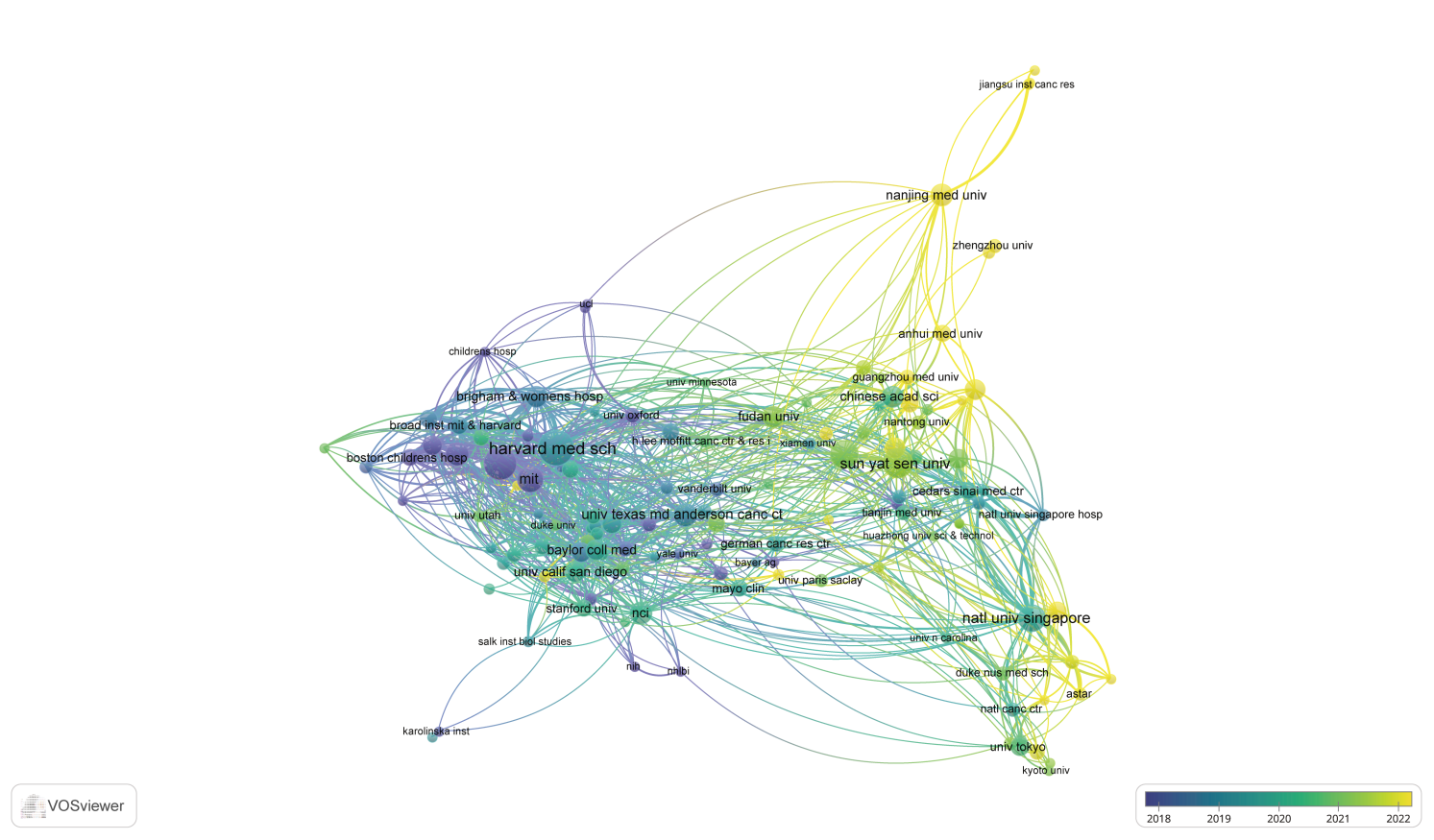


**Supplementary Figure 1.** The network visualization of institutions. The size of the circles is proportional to the number of collaborations of countries/regions/institutions in the cooperation network. The thickness of the lines indicates the strength of the connection between countries/regions/institutions.Node color reflects temporal activity,shifting from purple (earlier) to yellow (recent)

## Supplementary Tables

****Supplementary Table 1. Top 20 high-cited references in targeting super-Enhancers for cancer therapy research****

| Rank | Title | Journal | First author | Year | Citations |
| --- | --- | --- | --- | --- | --- |
| 1 | SELECTIVE INHIBITION OF TUMOR ONCOGENES BY DISRUPTION OF SUPER-ENHANCERS | CELL | LOVEN, JAKOB | 2013 | 2285 |
| 2 | TOX TRANSCRIPTIONALLY AND EPIGENETICALLY PROGRAMS CD8+ T CELL EXHAUSTION | NATURE | KHAN, OMAR | 2019 | 1094 |
| 3 | TRANSCRIPTIONAL ADDICTION IN CANCER | CELL | BRADNER, JAMES E. | 2017 | 869 |
| 4 | THE MECHANISMS BEHIND THE THERAPEUTIC ACTIVITY OF BET BROMODOMAIN INHIBITION | MOLECULAR CELL | SHI, JUNWEI | 2014 | 722 |
| 5 | TARGETING TRANSCRIPTION REGULATION IN CANCER WITH A COVALENT CDK7 INHIBITOR | NATURE | KWIATKOWSKI, NICHOLAS | 2014 | 697 |
| 6 | DISCOVERY AND CHARACTERIZATION OF SUPER-ENHANCER-ASSOCIATED DEPENDENCIES IN DIFFUSE LARGE B CELL LYMPHOMA | CANCER CELL | CHAPUY, BJOERN | 2013 | 619 |
| 7 | LIQUID-LIQUID PHASE SEPARATION IN DISEASE | ANNUAL REVIEW OF GENETICS, VOL 53 | ALBERTI, SIMON | 2019 | 614 |
| 8 | BRD4 AND CANCER: GOING BEYOND TRANSCRIPTIONAL REGULATION | MOLECULAR CANCER | DONATI, BENEDETTA | 2018 | 530 |
| 9 | RESPONSE AND RESISTANCE TO BET BROMODOMAIN INHIBITORS IN TRIPLE-NEGATIVE BREAST CANCER | NATURE | SHU, SHAOKUN | 2016 | 508 |
| 10 | ENHANCER HIJACKING ACTIVATES GFI1 FAMILY ONCOGENES IN MEDULLOBLASTOMA | NATURE | NORTHCOTT, PAUL A. | 2014 | 498 |
| 11 | CDK7 INHIBITION SUPPRESSES SUPER-ENHANCER-LINKED ONCOGENIC TRANSCRIPTION IN MYCN-DRIVEN CANCER | CELL | CHIPUMURO, EDMOND | 2014 | 496 |
| 12 | TRANSCRIPTION REGULATION BY THE MEDIATOR COMPLEX | NATURE REVIEWS MOLECULAR CELL BIOLOGY | SOUTOURINA, JULIE | 2018 | 422 |
| 13 | THE JAK-STAT PATHWAY AT 30:MUCH LEARNED, MUCH MORE TO DO | CELL | PHILIPS, RACHAEL L. | 2022 | 414 |
| 14 | ASCL1 AND NEUROD1 REVEAL HETEROGENEITY IN PULMONARY NEUROENDOCRINE TUMORS AND REGULATE DISTINCT GENETIC PROGRAMS | CELL REPORTS | BORROMEO, MARK D. | 2016 | 384 |
| 15 | NEUROBLASTOMA IS COMPOSED OF TWO SUPER-ENHANCER-ASSOCIATED DIFFERENTIATION STATES | NATURE GENETICS | VAN GRONINGEN, TIM | 2017 | 380 |
| 16 | CONVERGENCE OF DEVELOPMENTAL AND ONCOGENIC SIGNALING PATHWAYS AT TRANSCRIPTIONAL SUPER-ENHANCERS | MOLECULAR CELL | HNISZ, DENES | 2015 | 378 |
| 17 | BET PROTEINS AS TARGETS FOR ANTICANCER TREATMENT | CANCER DISCOVERY | STATHIS, ANASTASIOS | 2018 | 377 |
| 18 | PARTITIONING OF CANCER THERAPEUTICS IN NUCLEAR CONDENSATES | SCIENCE | KLEIN, ISAAC A. | 2020 | 371 |
| 19 | TARGETING TRANSCRIPTIONAL ADDICTIONS IN SMALL CELL LUNG CANCER WITH A COVALENT CDK7 INHIBITOR | CANCER CELL | CHRISTENSEN, CAMILLA L. | 2014 | 367 |
| 20 | CDK7-DEPENDENT TRANSCRIPTIONAL ADDICTION IN TRIPLE-NEGATIVE BREAST CANCER | CELL | WANG, YUBAO | 2015 | 366 |

****Supplementary Table2. Small molecule inhibitors targeting super-enhancers-driven transcription in cancers****

| Small molecule inhibitior | Target | Conditions | Clinical phase(Clinical Trial No.)/Reference |
| --- | --- | --- | --- |
| ABBV-744 | BRD2 | AML | [1] |
|  |  | Androgen receptor-positive prostate cancer | [2] |
| JQ1 | BRD4 | Prostate cancer | [3] |
|  |  | AML | [4] |
|  |  | Merkel cell carcinoma | [5] |
|  |  | MM | [6] |
|  |  | Osteosarcoma | [7] |
| iBET151 | BRD4 | Leukemia | [8] |
| I-BET-762 | BRD2/3/4 | NUT midline carcinoma | Phase I (NCT01587703) |
|  |  | Hematological malignancies | Phase I (NCT01943851) |
| I-BET-726 | BRD2/3/4 | Neuroblastoma tumor | [9] |
| BETd-246 | BRD2/3/4 | TNBC | [10] |
| OTX015 | BRD2/3/4 | AML | Phase I (NCT01713582) |
|  |  | Leukemia |  |
|  |  | DLBCL |  |
|  |  | ALL |  |
|  |  | MM |  |
|  |  | NUT midline carcinoma | Phase II (NCT02259114) |
|  |  | TNBC |  |
|  |  | NSCLC with rearranged ALK gene/fusion protein or KRAS mutation |  |
|  |  | CRPC |  |
|  |  | Pancreatic ductal adenocarcinoma |  |
|  |  | Glioblastoma multiforme | Phase II (NCT02296476) |
|  |  | AML | Phase I (NCT02698189) |
|  |  | NUT midline carcinoma | Phase I (NCT02698176) |
|  |  | TNBC |  |
|  |  | NSCLC |  |
|  |  | Castrate-resistant prostate cancer |  |
| CPI-0610 | BRD4 | Lymphoma | Phase I (NCT01949883) |
|  |  | MM | Phase I (NCT02157636) |
| THZ1 | CDK7 | OSCC | [11] |
|  |  | NSCLC | [12] |
|  |  | CRPC | [13] |
|  |  | MM | [14] |
|  |  | T-ALL | [15] |
|  |  | HCC | [16] |
|  |  | SCLC | [17] |
|  |  | PTCL | [18] |
| SY-1365 | CDK7 | Advanced solid tumors | Phase I (NCT03134638)/Syros Pharmaceuticals |
|  |  | Ovarian cancer |  |
|  |  | Breast cancer |  |
| SY-5609 | CDK7 | Advanced solid tumors | Recruiting/Syros Pharmaceuticals |
|  |  | Breast cancer |  |
|  |  | SCLC |  |
|  |  | Pancreatic cancer |  |
| Lee011 | CDK4/6 | Advanced solid tumors | Phase I (NCT01237236) |
|  |  | Lymphomas |  |
|  |  | Advanced solid tumors | Phase I (NCT01898845) |
|  |  | Malignant rhabdoid tumors,MRT | Phase I (NCT01747876) |
|  |  | Neuroblastoma |  |
|  |  | Advanced metastatic breast cancer | Phase III (NCT01958021) |
| PD0332991 | CDK4/6 | NSCLC | Phase II (NCT01291017) |
| AZD4573 | CDK9 | Relapsed or refractory haematological malignancies | Phase I (NCT03263637) |
| BAY1251152 | CDK9 | Hematologic neoplasm | Phase I (NCT02745743) |
| BI 894999 | CDK9 | Neoplasm | Phase I (NCT02516553) |
|  |  | NUT carcinoma |  |
| Cortistatain A | CDK8/19 | AML | [8] |
| THZ531 | CDK12/13 | Ewing sarcoma | [19] |
| SY-1425 | RARα | AML | Phase II (NCT02807558) / Syros Pharmaceuticals |
|  |  | Myelodysplastic syndrome |  |

AML: Acute myeloid leukemia; DLBCL: Diffuse large B cell lymphoma; ALL: Acute lymphoblastic leukemia; MM: Multiple myeloma; TNBC: Triple-negative breast cancer; OSCC: Oesophageal squamous cell carcinoma; NSCLC: Non-small cell lung cancer; CRPC: Castration-resistant prostate cancer; T-ALL: T-cell acute lymphoblastic leukemia; HCC: Hepatocellular carcinoma; SCLC: Small cell lung cancer; PTCL: Peripheral T-cell lymphomas.

**Reference**

1. Zhang L, Cai T, Lin X, Huang X, Bui MH, Plotnik JP, et al. (2021) Selective Inhibition of the Second Bromodomain of BET Family Proteins Results in Robust Antitumor Activity in Preclinical Models of Acute Myeloid Leukemia. Mol Cancer Ther 20:1809-19. <https://doi.org/10.1158/1535-7163.Mct-21-0029>

2. Faivre EJ, McDaniel KF, Albert DH, Mantena SR, Plotnik JP, Wilcox D, et al. (2020) Selective inhibition of the BD2 bromodomain of BET proteins in prostate cancer. Nature 578:306-10. <https://doi.org/10.1038/s41586-020-1930-8>

3. Wang L, Xu M, Kao CY, Tsai SY, Tsai MJ (2020) Small molecule JQ1 promotes prostate cancer invasion via BET-independent inactivation of FOXA1. J Clin Invest 130:1782-92. <https://doi.org/10.1172/jci126327>

4. Zuber J, Shi J, Wang E, Rappaport AR, Herrmann H, Sison EA, et al. (2011) RNAi screen identifies Brd4 as a therapeutic target in acute myeloid leukaemia. Nature 478:524-8. <https://doi.org/10.1038/nature10334>

5. Shao Q, Kannan A, Lin Z, Stack BC, Jr., Suen JY, Gao L (2014) BET protein inhibitor JQ1 attenuates Myc-amplified MCC tumor growth in vivo. Cancer Res 74:7090-102. <https://doi.org/10.1158/0008-5472.Can-14-0305>

6. Piddock RE, Marlein CR, Abdul-Aziz A, Shafat MS, Auger MJ, Bowles KM, et al. (2018) Myeloma-derived macrophage inhibitory factor regulates bone marrow stromal cell-derived IL-6 via c-MYC. J Hematol Oncol 11:66. <https://doi.org/10.1186/s13045-018-0614-4>

7. Wang H, Liu Z, Wang J, Hu F, Zhou Q, Wei L, et al. (2022) Superenhancers activate the autophagy-related genes Beclin1 and LC3B to drive metastasis and drug resistance in osteosarcoma. Front Med 16:883-95. <https://doi.org/10.1007/s11684-022-0919-0>

8. Pelish HE, Liau BB, Nitulescu, II, Tangpeerachaikul A, Poss ZC, Da Silva DH, et al. (2015) Mediator kinase inhibition further activates super-enhancer-associated genes in AML. Nature 526:273-6. <https://doi.org/10.1038/nature14904>

9. Wyce A, Ganji G, Smitheman KN, Chung CW, Korenchuk S, Bai Y, et al. (2013) BET inhibition silences expression of MYCN and BCL2 and induces cytotoxicity in neuroblastoma tumor models. PLoS One 8:e72967. <https://doi.org/10.1371/journal.pone.0072967>

10. Bai L, Zhou B, Yang CY, Ji J, McEachern D, Przybranowski S, et al. (2017) Targeted Degradation of BET Proteins in Triple-Negative Breast Cancer. Cancer Res 77:2476-87. <https://doi.org/10.1158/0008-5472.Can-16-2622>

11. Wang QY, Peng L, Chen Y, Liao LD, Chen JX, Li M, et al. (2020) Characterization of super-enhancer-associated functional lncRNAs acting as ceRNAs in ESCC. Mol Oncol 14:2203-30. <https://doi.org/10.1002/1878-0261.12726>

12. Wang J, Zhang R, Lin Z, Zhang S, Chen Y, Tang J, et al. (2020) CDK7 inhibitor THZ1 enhances antiPD-1 therapy efficacy via the p38α/MYC/PD-L1 signaling in non-small cell lung cancer. J Hematol Oncol 13:99. <https://doi.org/10.1186/s13045-020-00926-x>

13. Rasool RU, Natesan R, Deng Q, Aras S, Lal P, Sander Effron S, et al. (2019) CDK7 Inhibition Suppresses Castration-Resistant Prostate Cancer through MED1 Inactivation. Cancer Discov 9:1538-55. <https://doi.org/10.1158/2159-8290.Cd-19-0189>

14. Zhang Y, Zhou L, Bandyopadhyay D, Sharma K, Allen AJ, Kmieciak M, et al. (2019) The Covalent CDK7 Inhibitor THZ1 Potently Induces Apoptosis in Multiple Myeloma Cells In Vitro and In Vivo. Clin Cancer Res 25:6195-205. <https://doi.org/10.1158/1078-0432.Ccr-18-3788>

15. Kwiatkowski N, Zhang T, Rahl PB, Abraham BJ, Reddy J, Ficarro SB, et al. (2014) Targeting transcription regulation in cancer with a covalent CDK7 inhibitor. Nature 511:616-20. <https://doi.org/10.1038/nature13393>

16. Zhong L, Yang S, Jia Y, Lei K (2018) Inhibition of cyclin-dependent kinase 7 suppresses human hepatocellular carcinoma by inducing apoptosis. J Cell Biochem 119:9742-51. <https://doi.org/10.1002/jcb.27292>

17. Christensen CL, Kwiatkowski N, Abraham BJ, Carretero J, Al-Shahrour F, Zhang T, et al. (2014) Targeting transcriptional addictions in small cell lung cancer with a covalent CDK7 inhibitor. Cancer Cell 26:909-22. <https://doi.org/10.1016/j.ccell.2014.10.019>

18. Cayrol F, Praditsuktavorn P, Fernando TM, Kwiatkowski N, Marullo R, Calvo-Vidal MN, et al. (2017) THZ1 targeting CDK7 suppresses STAT transcriptional activity and sensitizes T-cell lymphomas to BCL2 inhibitors. Nat Commun 8:14290. <https://doi.org/10.1038/ncomms14290>

19. Jiang B, Jiang J, Kaltheuner IH, Iniguez AB, Anand K, Ferguson FM, et al. (2021) Structure-activity relationship study of THZ531 derivatives enables the discovery of BSJ-01-175 as a dual CDK12/13 covalent inhibitor with efficacy in Ewing sarcoma. Eur J Med Chem 221:113481. <https://doi.org/10.1016/j.ejmech.2021.113481>
